# Supplementary material for: Role of evolving sea surface temperature modes of variability in improving seasonal precipitation forecasts
Source: Commun Earth Environ. 2025 Apr 3;6(1):256. doi: 10.1038/s43247-025-02235-y (PMC11968401; doi:10.1038/s43247-025-02235-y)
Supplement: Supplementary file 2 — Supplementary Information [file 43247_2025_2235_MOESM2_ESM.pdf]

**Supplementary Information for**

# Role of evolving sea surface temperature modes of variability in improving seasonal precipitation forecasts

Agniv Sengupta,[a] Duane E. Waliser,[b,c] Michael J. DeFlorio,[a] Bin Guan,[b,c] Luca Delle Monache,[a] F. Martin Ralph[a]

[a] *Center for Western Weather and Water Extremes, Scripps Institution of Oceanography, University of California San Diego, La Jolla, California, USA*

[b] *Jet Propulsion Laboratory, California Institute of Technology, Pasadena, California, USA*

[c] *Joint Institute for Regional Earth System Science and Engineering, University of California Los Angeles, Los Angeles, California, USA*

*Corresponding author*: Agniv Sengupta, agsengupta@ucsd.edu

**Table of Contents**

- Page 2: Supplementary Tables 1-2
- Pages 3-10: Supplementary Figures 1-8
- Page 11: Supplementary Discussion
- Page 12: Supplementary References

**Supplementary Table 1. Description of the NMME models analyzed in the present study.**

| Modeling institution (country) | Model name | Number of ensemble members | Lead time (months) |
|---|---|---|---|
| National Centers for Environmental Prediction (United States) | CFSv2 | 24 | 9.5 |
| NASA Goddard Space Flight Center (United States) | GEOS-S2S | 4 | 8.5 |
| Canadian Centre for Climate Modeling and Analysis (Canada) | CanCM3 | 10 | 11.5 |
| Environment and Climate Change Canada (Canada) | CanCM4i | 10 | 11.5 |
| Environment and Climate Change Canada (Canada) | GEM5-NEMO | 10 | 11.5 |
| Geophysical Fluid Dynamics Laboratory (United States) | GFDL-CM2p5-FLOR-B01 | 12 | 11.5 |
| Geophysical Fluid Dynamics Laboratory (United States) | GFDL-SPEAR | 15 | 11.5 |

**Supplementary Table 2. Conceptual schematic of the *n*-fold cross-validation employed in the study for hindcast skill assessment.** The model is iteratively fit *n* times, each time using *n*-1 folds for model training (shaded in grey) and then evaluating model skill on the excluded unseen set (shaded in golden). The table highlights a seven-fold cross-validation over the period of 1949–2018. Here, the first decade of 1949–1958 is excluded in the first iteration (Test #1), and the model training only utilizes data from the remaining period of 1959–2018; the hindcast skill is then assessed over the independent decade. This process is iteratively carried forward and applied to each of the other seven 10-year periods in Tests #2–7.

| | | | | | | | |
|---|---|---|---|---|---|---|---|
| Test #1 | 1949-58 | 1959-68 | 1969-78 | 1979-88 | 1989-98 | 1999-08 | 2009-18 |
| Test #2 | 1949-58 | 1959-68 | 1969-78 | 1979-88 | 1989-98 | 1999-08 | 2009-18 |
| Test #3 | 1949-58 | 1959-68 | 1969-78 | 1979-88 | 1989-98 | 1999-08 | 2009-18 |
| Test #4 | 1949-58 | 1959-68 | 1969-78 | 1979-88 | 1989-98 | 1999-08 | 2009-18 |
| Test #5 | 1949-58 | 1959-68 | 1969-78 | 1979-88 | 1989-98 | 1999-08 | 2009-18 |
| Test #6 | 1949-58 | 1959-68 | 1969-78 | 1979-88 | 1989-98 | 1999-08 | 2009-18 |
| Test #7 | 1949-58 | 1959-68 | 1969-78 | 1979-88 | 1989-98 | 1999-08 | 2009-18 |

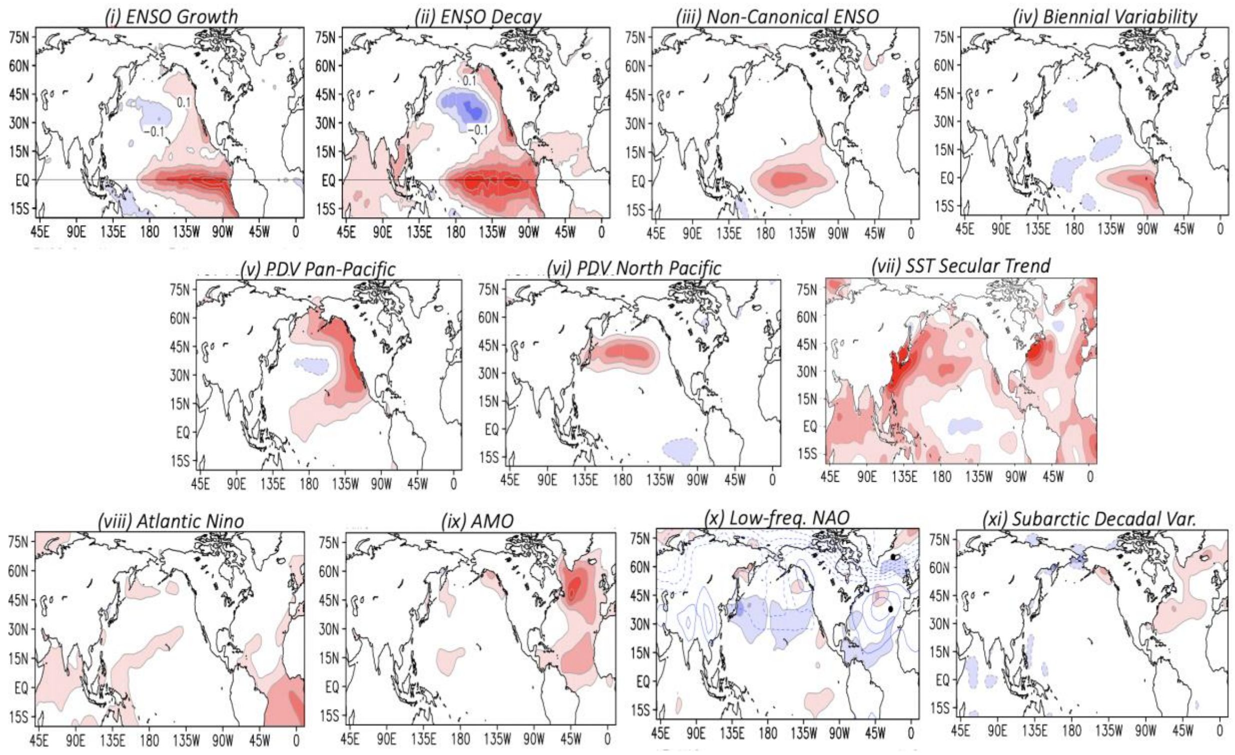


**Supplementary Fig. 1. Modes of SST variability informing the seasonal precipitation prediction.** These modes are obtained from an extended-EOF analysis of observed SST anomalies over the 20°S–80°N, 0°–360° domain, following Nigam et al.[1]. The analysis yields four modes comprising different flavors of ENSO variability (top panel), two modes of Pacific decadal variability as well as the secular trend (middle panel), and four modes of variability in the Atlantic (bottom panel). Individual panels depict the mature-phase spatial structure of each SST mode. Red and blue shading denotes associated positive and negative SST anomalies respectively. The contour interval and shading threshold is 0.1°C.

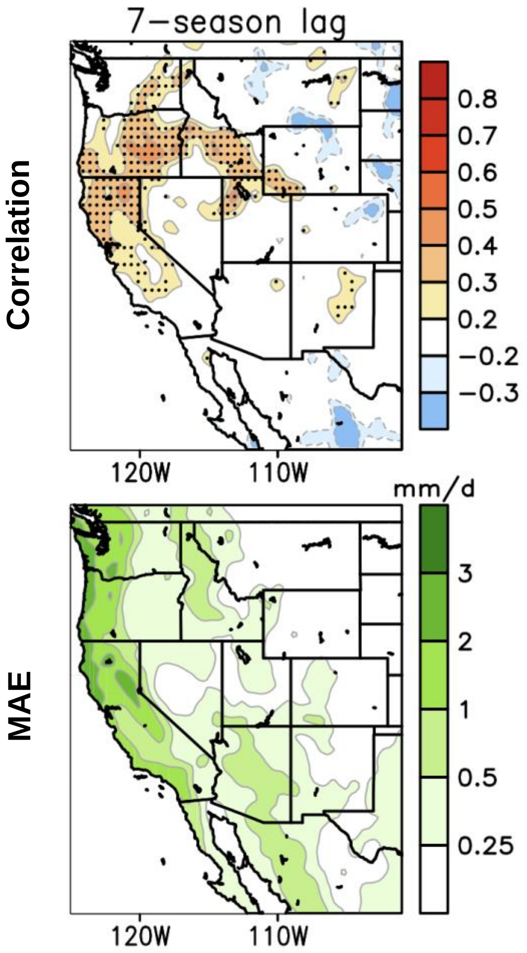


**Supplementary Fig. 2. Sensitivity test highlighting the influence of antecedent, multi-season lagged predictors.** The hindcast skill scores are obtained from *n*-fold cross-validation analyses and displayed as a function of the number of temporal lags employed in the MLMS–SST model, which in this case, is 7 seasons preceding the time of forecast issuance. Skill is depicted here in terms of anomaly correlations (top panel) as well as mean absolute error (MAE in mm $day^{-1}$; bottom panel) between the model-predicted and observed winter precipitation anomalies. The hindcast period of assessment is 1969–2018. The correlations that are statistically significant at the 95% confidence level are stippled in black.

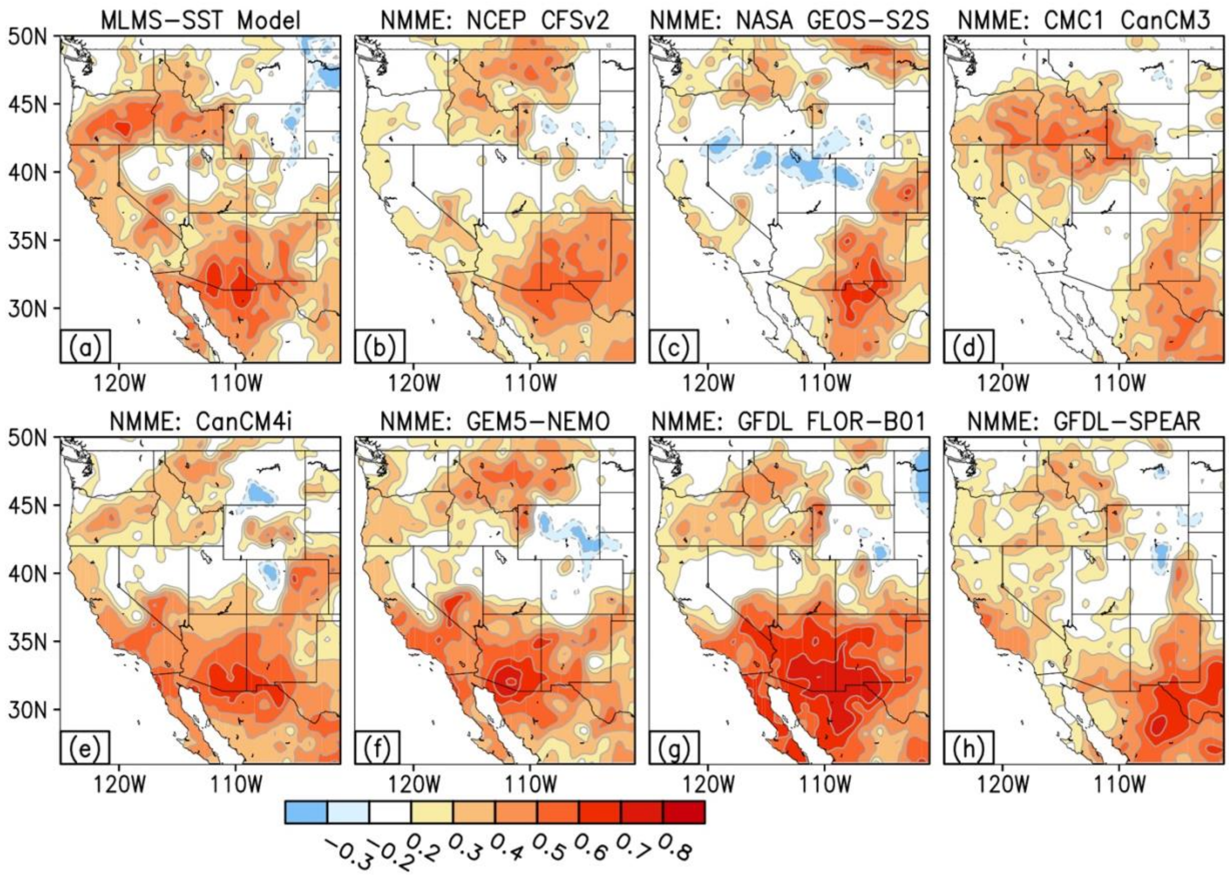


**Supplementary Fig. 3. Hindcast skill assessment of MLMS-SST and NMME dynamical models.** The dynamical models assessed here (panels **b–h**) include the NCEP-CFSv2, NASA GEOS-S2S, CMC CanCM3, CanCM4i, GEM5-NEMO, GFDL FLOR-B01, and GFDL-SPEAR, with hindcasts initialized in October for the November through March winter season. Skill is depicted here in terms of correlations between the observed and individual model-predicted precipitation anomalies for the common overlapping period of available hindcasts —winters 1982-83 through 2010-11 (except GFDL-SPEAR, available only from winter 1991-92). The correlation values are contoured and shaded in red at intervals of 0.1 when ≥ +0.2, and in blue when ≤ -0.2.

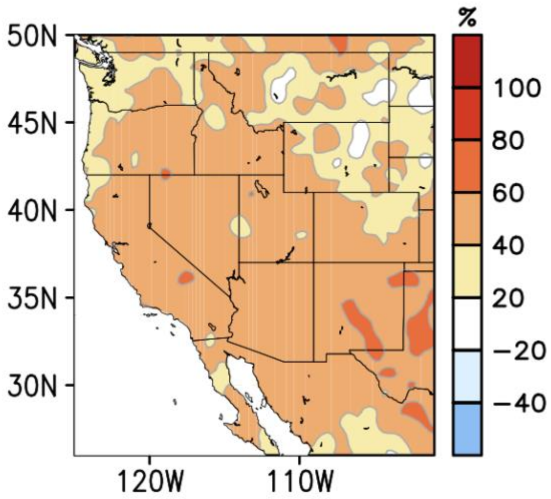


**Supplementary Fig. 4. Skill comparison with statistical benchmark model.** Skill score is assessed here in terms of percentage improvement over reference forecast following Murphy[2], as follows: $SS = \left[1 - \frac{A}{A_{ref}}\right] * 100\%$, where A represents the accuracy in terms of mean-squared-error (MSE) of the forecast from the MLMS–SST model; $A_{ref}$ is the accuracy of the reference forecast, given here by the persistence forecast. In case of a perfect model hindcast, the MSE will be equal to zero, i.e., $A = A_{perfect}$, yielding a skill score of 100%.

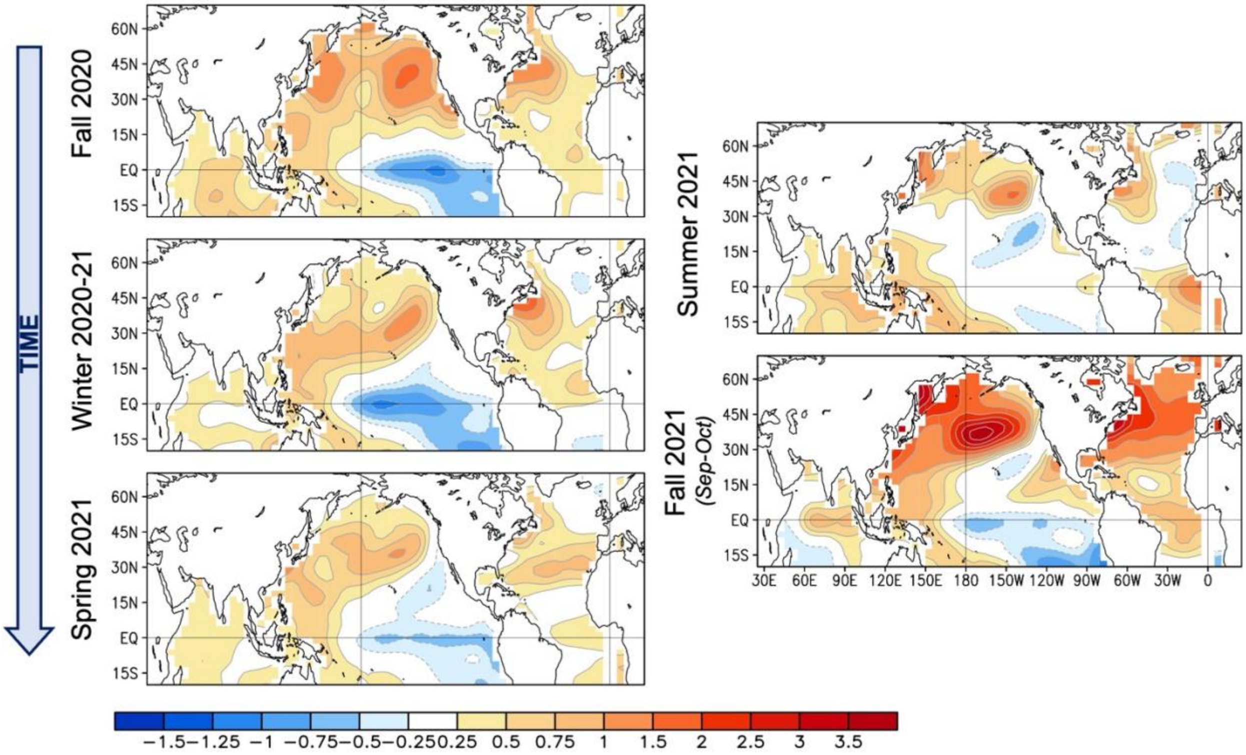


**Supplementary Fig. 5. The multi-season SST anomaly fields informing the NDJFM 2021-22 seasonal precipitation forecast.** The observed SST anomalies are shown over five seasons, from the Fall of 2020 to the Fall of 2021 (comprising September and October SSTs only). Red and blue shading denotes positive/warmer-than-normal and negative/colder-than-normal SSTs respectively.

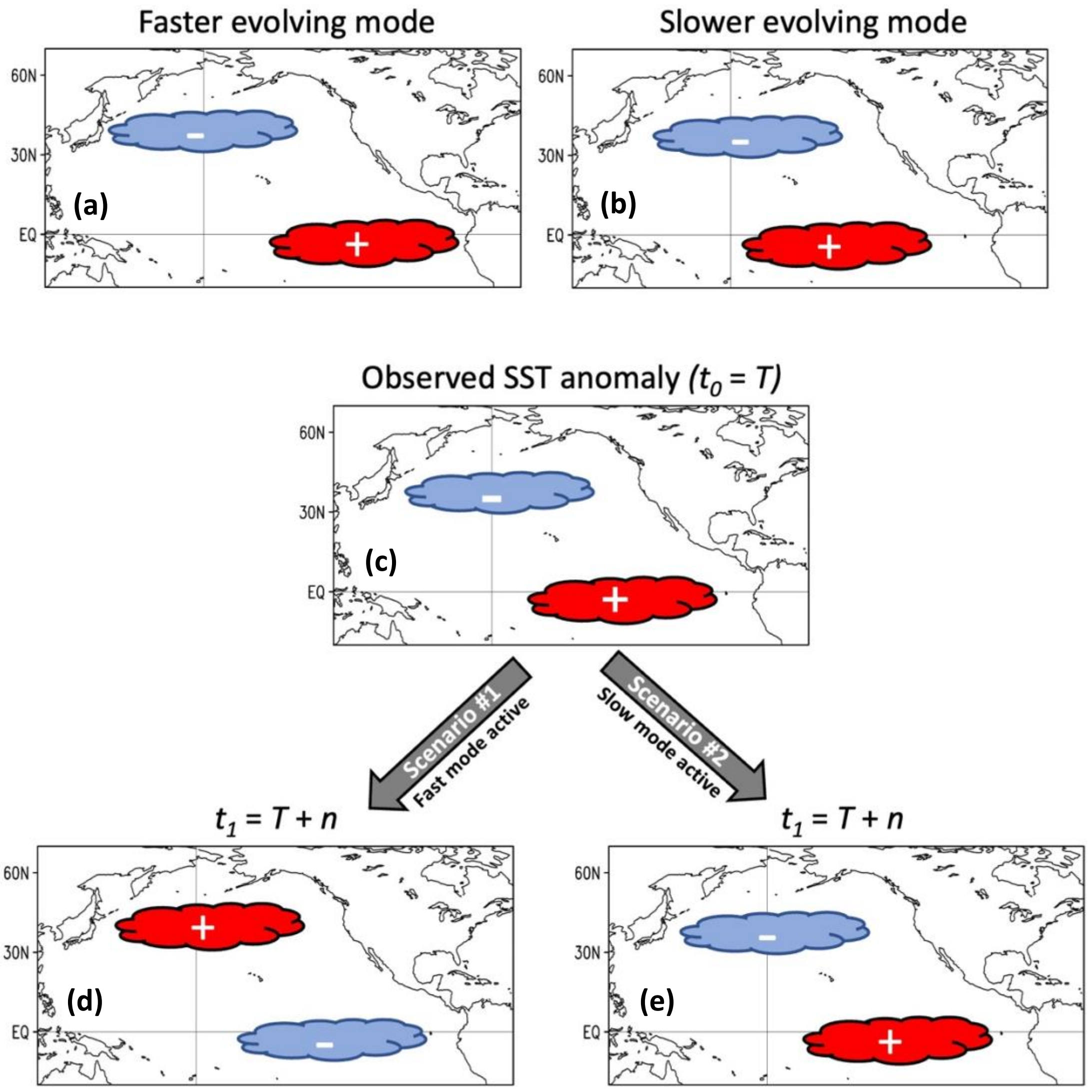


**Supplementary Fig. 6. Hypothetical case study demonstrating the need to utilize multiple past seasons rather than just the preceding season.** Panels **a** and **b** depict the SST anomaly spatial footprint associated with two hypothetical modes: a high-frequency or faster-evolving mode, and another low-frequency or slowly evolving mode. Panel **c** shows the observed SST anomaly field at time $t_0 = T$. Panels **d** and **e** illustrate two possible scenarios at a future time, $t_1 = T + n$, based on which of the two SST modes is active. Red and blue shading denotes positive/warmer-than-normal and negative/colder-than-normal SSTs respectively.

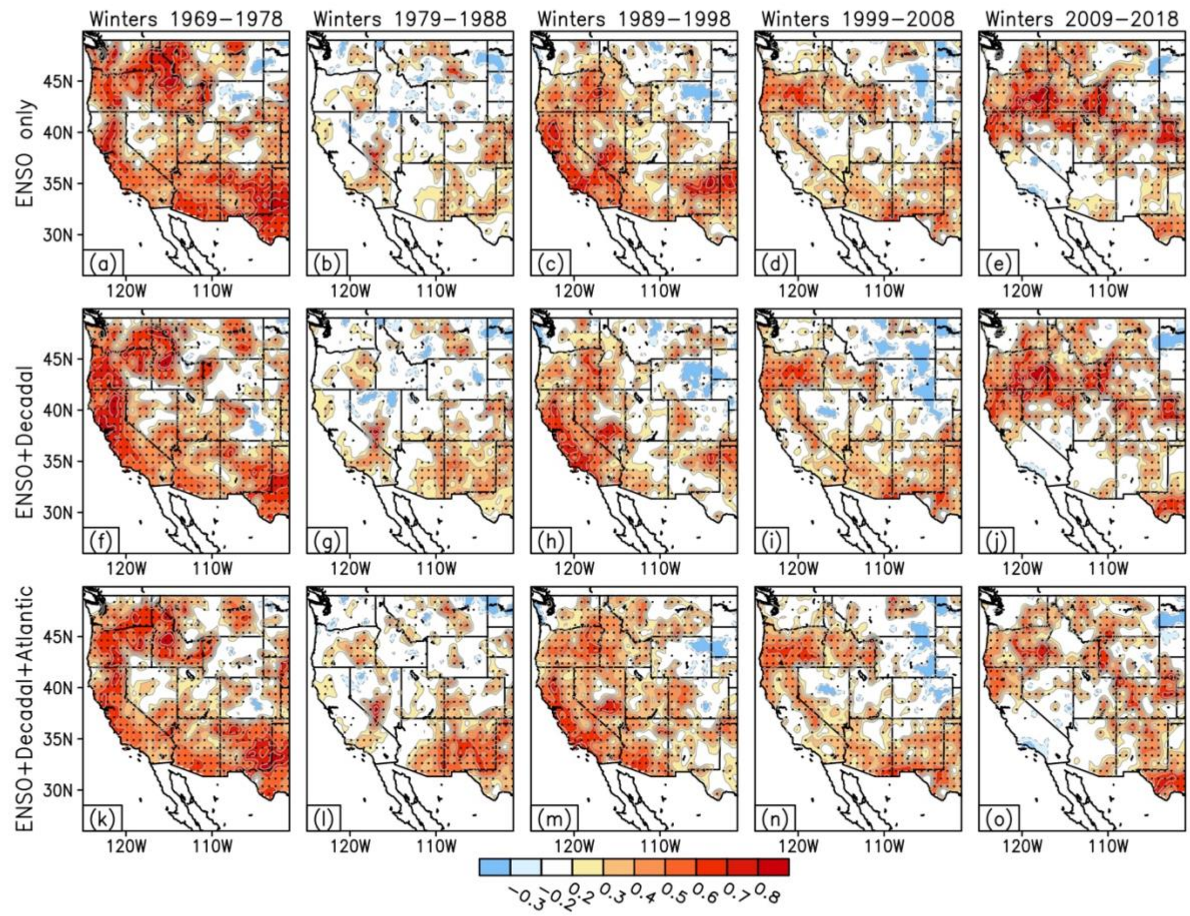


**Supplementary Fig. 7. Hindcast skill of the MLMS–SST model across the western U.S. when performing model training with NOAA CPC Unified precipitation observations instead of GPCC version 2020 dataset.** Cross-validation analyses over independent hindcast periods yield correlations between the observed and model-predicted winter precipitation anomalies. Panels **a–e** denote skill scores obtained when only ENSO modes of variability are considered in the predictor set, whereas subsequent panels depict skill with additional contributions from the secular trend and decadal variability modes in the Pacific (panels **f–j**) and in the Atlantic (panels **k–o**). Correlations that are statistically significant at the 95% confidence level are stippled in black. The correlation values are contoured and shaded in red at intervals of 0.1 when ≥ +0.2, and in blue when ≤ -0.2.

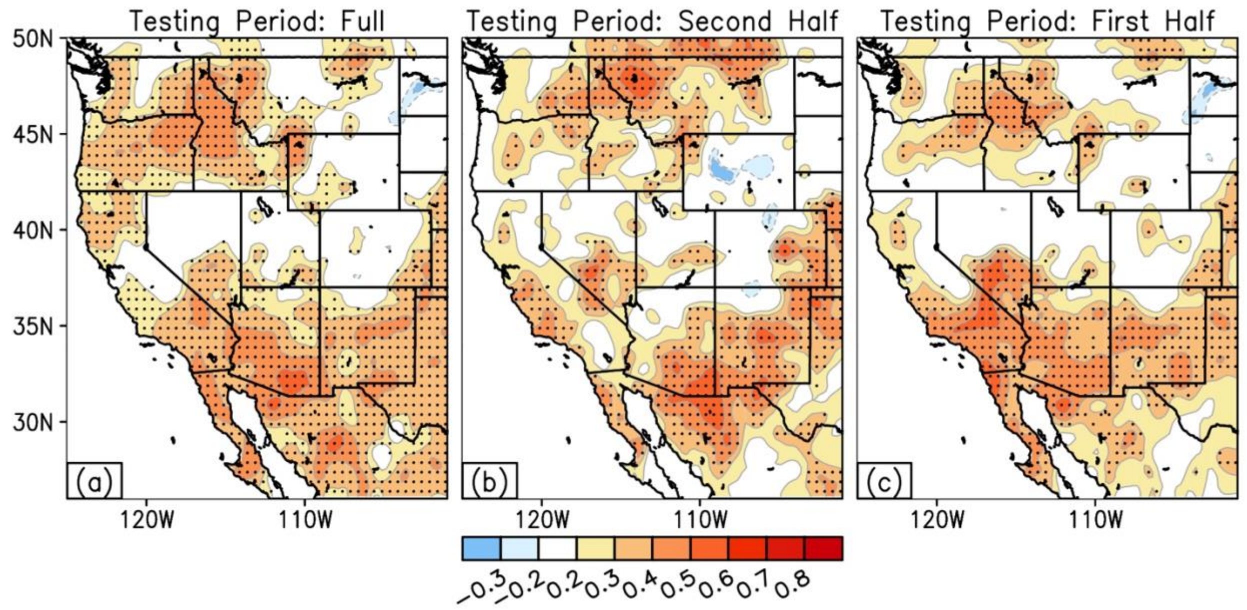


**Supplementary Fig. 8. Additional precipitation hindcast skill assessments of the MLMS-SST model (a)** performed over the full period of analysis, **(b)** by training on the first half and testing on the independent second half of the record, and **(c)** by training on the second half and testing on the independent first half of the record. The primary extended-EOF analysis is modified in (b) and (c) to leverage predictors extracted solely from the corresponding half of the data. Hindcast skill is depicted here using correlations between the observed and model-predicted winter precipitation anomalies. Correlations that are statistically significant at the 95% confidence level are stippled in black. The correlation values are contoured and shaded in red at intervals of 0.1 when ≥ +0.2, and in blue when ≤ -0.2.

## Supplementary Discussion

## Additional analyses on precipitation hindcast skill

In the main article, we investigated the hindcast skill of the MLMS−SST model over the western United States during the boreal winter (November to March) using a *n*-fold cross-validation approach and reported skill over the recent five decades ranging from 1969 to 2018. Additionally, here we perform split cross-validation analyses by modifying the primary extended-EOF analysis to use SST data only for one-half of the data record and then evaluating seasonal precipitation forecasts over the independent second half, presented as Supplementary Figure S8. In other words, no SST data from the independent precipitation skill assessment period is used to extract the extended-EOF modes that inform the model training. The model training solely relies on one-half of the period of analysis, i.e., either 1949-1983 or, 1984-2018, to learn the SST modes (predictors) and precipitation (predictand) relationship. The motivation for these analyses was to ensure that the model does not exhibit elevated skill due to sampling variability.

The hindcast skill assessment maps reveal similar swaths of areas in the western U.S. with statistically significant correlations across the three assessment periods, e.g., over the Pacific Northwest, California, Lower Colorado River basin, and parts of the Great Basin. However, it is important to note a couple of caveats in this modified approach, where the model training does not utilize half of the available data for seasonal forecasting, which is an inherently data-limited problem. First, the relatively short period of the modified SST analysis may be inadequate to skillfully extract the decadal-multidecadal modes of SST variability. The SST observed record is already fairly limited and affords less than two cycles of multidecadal variability. Hence, restricting it even further may lead to a suboptimal extraction of modes of decadal-multidecadal variability. Second, in the curtailed analysis (e.g., when utilizing training data only up to 1983), the secular trend (related to the oceanic component of global warming) might not emerge as the leading mode of variability as in the case of primary extended-EOF analysis that leverages the full data record. As we enter a warmer world, a robust characterization and inclusion of the influence of secular warming on regional precipitation changes will be increasingly important.

## Supplementary References


1. Nigam, S., Sengupta, A. & Ruiz-Barradas, A. Atlantic–Pacific links in observed multidecadal SST variability: is the Atlantic multidecadal oscillation's phase reversal orchestrated by the Pacific decadal oscillation? *J. Clim.* **33**, 5479–5505 (2020).
2. Murphy, A. H. Skill scores based on the mean square error and their relationships to the correlation coefficient. *Mon. Weather Rev.* **116**, 2417–2424 (1988).
